# Secondhand smoke in combination with ambient air pollution exposure is associated with increasedx CpG methylation and decreased expression of *IFN-γ* in T effector cells and *Foxp3* in T regulatory cells in children

**DOI:** 10.1186/1868-7083-4-17

**Published:** 2012-09-25

**Authors:** Arunima Kohli, Marco A Garcia, Rachel L Miller, Christina Maher, Olivier Humblet, S Katharine Hammond, Kari Nadeau

**Affiliations:** 1 Department of Pediatric Allergy and Immunology, Stanford University, 269 Campus Drive, Stanford, CA, 94305, USA; 2 Division of Pulmonary, Allergy and Critical Care Medicine, Department of Medicine, Columbia University Medical Center, PH8E, 630 West 168th Street, New York, NY, 10032, USA; 3 Division of Pediatric Allergy and Immunology, Department of Pediatrics, Columbia University Medical Center, PH8E, 630 West 168th Street, New York, NY, 10032, USA; 4 Division of Environmental Health Sciences, University of California, 50 University Hall, Berkeley, CA, 94720, USA

**Keywords:** Secondhand smoke, Ambient air pollution, IFN-γ, Foxp3, Methylation, Epigenetic regulation, Pediatrics, T effectors, T regulatory cells

## Abstract

**Background:**

Secondhand smoke (SHS) and ambient air pollution (AAP) exposures have been associated with increased prevalence and severity of asthma and DNA modifications of immune cells. In the current study, we examined the association between SHS and AAP with DNA methylation and expression of interferon-gamma (*IFN-γ*) and forkhead box protein 3 (*Foxp3*) in T cell populations.

**Methods:**

Subjects 7–18 years old were recruited from Fresno (high AAP; n = 62) and Stanford, CA (low AAP; n = 40) and divided into SHS-exposed (Fresno: n = 31, Stanford: n = 6) and non-SHS-exposed (nSHS; Fresno: n = 31, Stanford: n = 34) groups. T cells purified from peripheral blood were assessed for levels of DNA methylation and expression of *IFN-γ* (in effector T cells) or *Foxp3* (in regulatory T cells).

**Results:**

Analysis showed a significant increase in mean % CpG methylation of *IFN-γ* and *Foxp3* associated with SHS exposure (*IFN-γ*: FSHS 62.10%, FnSHS 41.29%, *p* < 0.05; SSHS 46.67%, SnSHS 24.85%, *p* < 0.05; *Foxp3*: FSHS 74.60%, FnSHS 54.44%, *p* < 0.05; SSHS 62.40%, SnSHS 18.41%, *p* < 0.05) and a significant decrease in mean transcription levels of both genes (*IFN-γ*: FSHS 0.75, FnSHS 1.52, *p* < 0.05; SHS 2.25, nSHS 3.53, *p* < 0.05; *Foxp3*: FSHS 0.75, FnSHS 3.29, *p* < 0.05; SSHS 4.8, SnSHS 7.2, *p* < 0.05). AAP was also associated with hypermethylation (*IFN-γ*: FSHS vs. SSHS, *p* < 0.05; FnSHS vs. SnSHS, *p* < 0.05; *Foxp3*: FSHS vs. SSHS, *p* < 0.05; FnSHS vs. SnSHS, *p* < 0.05) and decreased transcription of both genes (*IFN-γ*: FSHS vs. SSHS, *p* < 0.05; FnSHS vs. SnSHS, *p* < 0.05; *Foxp3*: FSHS vs. SSHS, *p* < 0.05; FnSHS vs. SnSHS, *p* < 0.05). Average methylation between AAP- and SHS-only exposures was not significantly different (*IFN-γ*: *p* = 0.15; *Foxp3*: *p* = 0.27), nor was *Foxp3* expression (*p* = 0.08); *IFN-γ* expression was significantly decreased in AAP-only subjects (*p* < 0.05).

**Conclusions:**

Exposures to SHS and AAP are associated with significant hypermethylation and decreased expression of *IFN-γ* in Teffs and *Foxp3* in Tregs. Relative contributions of each exposure to DNA modification and asthma pathogenesis warrant further investigation.

## Background

Exposure to secondhand smoke (SHS) is associated with a number of adverse health effects, including cancer, cardiovascular disease, and increased frequency and severity of respiratory disease [1]. Per the US Surgeon General’s 2006 report, any exposure to SHS results in increased health risks [1]. The World Health Organization (WHO) estimates that 22% of women and 35% of men in developed countries smoke cigarettes [2]; in the US alone, it has been estimated that over 126 million nonsmokers are exposed to SHS [3]. Most SHS exposure occurs in the home, thus making children the most vulnerable population: the WHO estimates that 40% of children worldwide are exposed to SHS at home [2,3].

Of particular interest is SHS’s role in the development and exacerbation of asthma and allergic disease. Asthma is the most common chronic disease in children [4], and numerous studies have associated both pre- and postnatal SHS exposure with increased life-long risk of asthma, with evidence of this association passing between generations [1,3,5-7]. In fact, SHS exposure has been estimated to cause asthma symptoms in 200,000 to 1 million children, contributing to anywhere from 8,000 to 26,000 new cases each year [3]. Many asthmatic children are also atopic (that is, display allergic sensitization to aeroallergens). Atopy and allergic rhinitis also have been associated with parental smoking and SHS exposure [5], though the relationship between SHS and allergic rhinitis is unclear [2].

SHS is not the only environmental exposure implicated in these diseases; ambient air pollution (AAP) is also known to contribute. AAP includes such compounds as polycyclic aromatic hydrocarbons (PAH), particulate matter with a diameter ≤ 2.5 μm (PM_2.5_), particulate matter with a diameter ≤ 10 μm (PM_10_), elemental carbon, and ozone, and is also known to be associated with increased prevalence and severity of various diseases and adverse health effects, including asthma and allergies [8,9]. Again, children are particularly vulnerable.

Because smoking (and consequently SHS exposure) and AAP are so widespread, understanding the mechanisms by which SHS in combination with AAP impacts human health is crucial, especially with regard to children. A recent study asked which environmental exposure (acute SHS exposure vs. AAP exposure, using cotinine to measure SHS exposure and PM_2.5_ to measure AAP exposure) played more of a role in children’s respiratory health; their clinical data suggested that SHS modulates the acute impact of PM_2.5_ exposure on asthma in children, implying that acute SHS exposure has a greater effect on respiratory health than does acute AAP exposure [10]. Our group has published data on ambient air pollution exposure (specifically focusing on PAH) and its effects on asthma and regulatory T cell function [4]. Exposure to both mainstream cigarette smoking and SHS has been shown to have sustained impacts on health [3,5,11-13]. Most strikingly, these effects have been shown to pass between generations [6], suggesting that one way SHS may impact human health is via epigenetic alteration. *In utero* smoke exposure from maternal smoking has been associated with changes in both global DNA methylation and methylation of specific genes [11,12,14,15]. Moreover, various components of AAP that are shared with SHS (for example, PAH) have been found to impact DNA methylation [3,11,16,17]. Therefore, we chose to study the mechanisms underlying the combined effects of SHS and AAP on specific immune cells that are associated with health outcomes such as asthma.

We chose to focus our mechanistic studies on the epigenetic regulation of interferon-gamma (*IFN-γ*), a Th1 cytokine that is a known negative regulator of allergic responses [18,19], and forkhead box transcription factor 3 (*Foxp3*), which is essential to the development and function of T regulatory cells (Tregs) [4]. Transcription and expression of both these genes are influenced by methylation of cytosine nucleotide neighboring guanine (CpG) sites. *IFN-γ* expression is inversely related to the degree of methylation of specific CpG sites within the gene [20-22]. The CpG site in the promoter of *IFN-γ* is hypomethylated in Th1 cells and hypermethylated in Th2 cells (both subsets of T effector cells, or Teffs), the latter of which are associated with pro-allergic and -inflammatory responses [23,24]. *Foxp3* is also negatively regulated by CpG methylation of its transcriptional regulatory regions [25], requiring complete demethylation of its CpG sites for stable expression [26]. In Tregs, which require stable *Foxp3* expression to function normally [4], the gene’s promoter is completely demethylated [27].

Studies have shown that CpG methylation of regulatory elements of both *IFN-γ* and *Foxp3* is affected by environmental exposures. Liu *et al*. [28] found that a combination of diesel exhaust particle and allergen exposure in an allergic mouse model led to hypermethylation of three CpG sites in the *IFN-γ* promoter, as well as increased IgE production. Kwon *et al*. [23] showed that stimulation of CD4^+^ cells from adult asthmatic patients sensitized to dust mite with phytohemagglutinin (PHA) altered the methylation of various CpG sites surrounding the promoter of the *IFN-γ* gene, though no consistent pattern emerged across the sites. Nadeau *et al*. [4] found that increased exposure to AAP (specifically, PAH) was associated with hypermethylation of *Foxp3* as well as impaired Treg function and increased rates and severity of asthma.

We examined the association between SHS in combination with AAP (specifically, PM_2.5_, PM_10,_ PAH, and ozone, all of which have been shown to impact respiratory symptoms as well as T cell ratios and levels of cytokines such as IFN-γ, IL-4, and IL-17 [29-33]), and the epigenetic regulation of *IFN-γ* in Teffs, and *Foxp3* in Tregs, of children under 18 years. AAP-exposed subjects were recruited from Fresno, California, which has very high rates of ambient air pollution and asthma [4]. Control subjects (subjects not exposed to high levels of AAP) were recruited from Palo Alto, California (referred to as the Stanford cohort in this paper), which has relatively low rates of ambient air pollution (Table 1).

**Table 1:** Comparison of ambient air pollutant concentrations between Fresno and Palo Alto, CA, based on California Air Resources Board (CARB) compliance monitoring for 2011

**Pollutant**	**Location of compliance monitor**	
	Redwood City*	**First Street, Fresno**
**PAHs, annual average (ng/m**^**3**^**)**	84	1,068
**PM**_**2.5**_**, annual average (μg/m**^**3**^**)**	Insufficient data	15.9
**PM**_**2.5**_**, 24-h maximum (μg/m**^**3**^**)**	24.0	78.5
**PM**_**10**_**, annual average (μg/m**^**3**^**)**	Insufficient data	29.6
**PM**_**10**_**, 24-h high (μg/m**^**3**^**)**	Insufficient data	99.5
**O**_**3**_**, highest 8-h average (ppb)**	62	97
**O**_**3**_**, number of days > state 1-h standard**	0	14
**O**_**3**_**, number of days > state 8-h standard**	0	54

PM_2.5_, particulate matter with aerodynamic diameter ≤ 2.5 μm; PM_10_, particulate matter with aerodynamic diameter ≤10 μm; PAH, polycyclic aromatic hydrocarbon; O_3_, ozone.

*Redwood City is the nearest compliance monitor, within 4.7 km of the Palo Alto residences of the Stanford cohort. All Fresno subjects live within a circle with the First Street monitor as its center and a radius of 20 km.

Drawing on evidence from previous studies examining the impact of environmental exposures on these genes, we hypothesized that exposure to SHS and AAP in children might increase methylation of regulatory elements in *IFN-γ* and *Foxp3* in Teffs and Tregs, respectively, and that this would lead to decreased expression of these genes in their respective cell types, with this effect more prominent in subjects exposed to SHS in combination with AAP vs. in subjects exposed to one or the other.

## Results

### General subject characteristics

Demographics of subjects are summarized in Table 2. Subjects from the Fresno cohort were divided into two groups based on SHS exposure: subjects with secondhand smoke exposure (FSHS) (n = 31) and subjects without secondhand smoke exposure (FnSHS) (n = 31). Subjects from the Stanford cohort (n = 40) were also divided into two control groups: thirty-four subjects without SHS exposure (SnSHS), and six with SHS exposure (SSHS). Mean ages within and between the Fresno and Stanford subject groups differed significantly (summarized in Table 2; *P* < 0.05). Male-to-female ratios within and between groups did not differ significantly (Table 2). IgE levels were significantly elevated in FSHS subjects compared to FnSHS subjects and SnSHS subjects, but not compared to SSHS subjects (FSHS mean ± SEM 76 ± 15 kU/L, FnSHS mean: 13 ± 7, *P* < 0.05; SnSHS mean: 11 ± 5 kU/L, *P* < 0.05; SSHS mean: 23 ± 9 kU/L, *P* = 0.14). IgE levels did not differ significantly between FnSHS subjects and SnSHS subjects, nor did they differ significantly between SSHS and SnSHS subjects. Allergic rhinitis prevalence did not differ significantly between any of the groups.

**Table 2:** Demographics of subjects

	Fresno SHS*	**Fresno nSHS**	Stanford SHS*	**Stanford nSHS**
	**(n = 31)**	**(n = 31)**	**(n = 6)**	**(n = 34)**
**Age, years**	14.13 ± 0.36 (range 11 to 18)	15.23 ± 0.28 (range 12 to 18)	16.17 ± 1.45 (range 9 to 18)	11.85 ± 3.13 (range 7 to 18)
**Gender, male/female**	15/16	20/11	2/4	17/17
**Number with allergic rhinitis**	9 (29.0%)	9 (29.0%)	2 (33%)	6 (17%)
**IgE levels, kU/L**	76 ± 15	13 ± 7	23 ± 9	11 ± 5
**Urine cotinine, ng/mL**	12.36 ± 6.86	0 ± 0	n/a	n/a
	(n = 16)	(n = 6)		
**Number of subjects with positive urine cotinine levels (> 7 ng/mL)**	5 (31.25%)	0 (0%)	n/a	n/a
	(n = 16)	(n = 6)		

*Two subjects each in both the Fresno and Stanford SHS groups were themselves smokers in addition to being exposed to SHS. SHS, secondhand smoke; nSHS, not exposed to secondhand smoke; kU/L, kilounits per liter; ng/mL, nanograms per milliliter. Means are reported ± standard error of the mean (SEM).

As proof of concept that our screening process to distinguish between subjects who were exposed to SHS and those who were not was accurate, subjects from both Fresno groups were chosen randomly for analysis of urine cotinine content via ELISA (FSHS n = 16, FnSHS n = 6). Urine cotinine levels were significantly greater in SHS-exposed subjects vs. non-SHS-exposed subjects, but the percentage of FSHS subjects showing urine cotinine levels above the threshold value of 7 ng/mL was not significant (FSHS mean cotinine ± SEM: 12.36 ng/mL ± 6.86, FnSHS mean: 0 ± 0, *P* < 0.05; FSHS above 7 ng/mL: 31.25%, FnSHS above 7 ng/mL: 0%, *P* = 0.27). Urinary cotinine was not measured for the Stanford subjects.

### *IFN-γ* in Teffs

**Figure 1:**
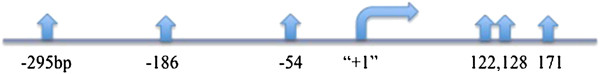
**Diagrammatic representation of the *IFN-γ *promoter region [J00219: GenBank].** Vertical arrows indicate cytosine neighboring guanine (CpG) dinucleotide sequences that can be potentially methylated. The position of each CpG dinucleotide site is illustrated in relation to the transcription start, which is indicated as +1 bp (base pair).

**Figure 2:**
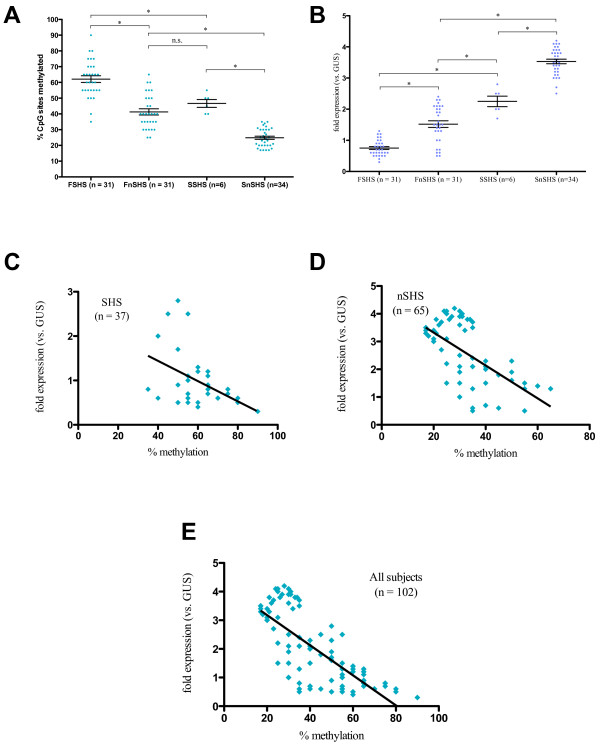
**Methylation and expression of *IFN-γ *in T effector cells (Teffs) of all subjects. A**. Comparison of overall methylation of examined cytosine nucleotide neighboring guanine (CpG) sites in *IFN-γ* in Teffs between Fresno subjects exposed to secondhand smoke (SHS) (FSHS) (n = 31), Fresno subjects not exposed to SHS (FnSHS) (n = 31), Stanford subjects exposed to SHS (SSHS) (n = 6), and Stanford subjects who were not exposed (SnSHS) (n = 34) groups. Methylation is reported as percent methylated CpG sites out of 6 total examined CpG sites (Figure 1); the cutoff for positive methylation at each site was set at 70% of all sequencing reactions. Error bars represent standard error of the mean (SEM). **B**. Comparison of expression of *IFN-γ* in Teffs between FSHS, FnSHS, SSHS, and SnSHS groups. Expression is reported as fold expression relative to the expression of the housekeeping gene beta-glucuronidase (*GUS*). Error bars represent SEM. **C**. Linear regression analysis of *IFN-γ* expression in Teffs vs. % methylation of *IFN-γ* from all SHS subjects. **D**. Linear regression analysis of *IFN-γ* expression in Teffs vs. % methylation of *IFN-γ* from all nSHS subjects. **E**. Linear regression analysis of *IFN-γ* expression in Teffs vs. % methylation of *IFN-γ* from all subjects. FSHS, Fresno group exposed to secondhand smoke; FnSHS, Fresno group not exposed to secondhand smoke; SSHS, Stanford group exposed to secondhand smoke; SnSHS, Stanford group not exposed to secondhand smoke; n.s., not significant. *P < 0.05.

Methylation of 6 CpG sites in the promoter region of the *IFN-γ* locus was analyzed via pyrosequencing (Figure 1). Sites were considered methylated only if they were methylated in 70% or more of all pyrosequencing reactions. Percent methylation for each individual was calculated by dividing the number of methylated CpG sites by the total number of CpG sites examined. We compared the average percent methylation of *IFN-γ* between Teffs of FSHS, FnSHS, SSHS, and SnSHS subjects. Two-way analysis of variance (ANOVA) found a significant source of variation associated with SHS exposure (35.12% of total variation, *P* < 0.05) and with AAP exposure (19.63% of total variation, *P* < 0.05), with no significant interaction effect (0.02% of total variation, *P* = 0.84). *T*-tests showed the average methylation of *IFN-γ* was significantly greater in the Teffs from FSHS subjects than in the Teffs from FnSHS subjects (Figure 2A; FSHS mean ± SEM: 62.10% ± 2.12%, FnSHS mean: 41.29% ± 1.94%, *P* < 0.05; Hedge’s *g*: 1.82, 95% CI [1.60, 2.10]), as well as in the Teffs from SSHS subjects vs. SnSHS subjects (Figure 2A; SSHS mean ± SEM: 46.67% ± 2.47%, SnSHS mean: 24.85% ± 0.99%, *P* < 0.05; Hedge’s *g*: 4.01, 95% CI [3.67, 4.5]). *IFN-γ* average methylation in Teffs was also significantly larger in FSHS vs. SSHS subjects (Figure 2A; *P* < 0.05; Hedge’s *g*: 1.35, 95% CI [1.17, 1.60]), but did not differ significantly between FnSHS and SSHS subjects (Figure 2A; *P* = 0.16). The effect size of AAP alone (FnSHS vs. SnSHS) was calculated to be 1.90 (95% CI [1.62, 2.23]), and the combined effect size of SHS and AAP exposure on *IFN-γ* methylation (FSHS vs. SnSHS) was calculated to be 4.01 (95% CI [3.67, 4.5]).

Transcription of *IFN-γ* in Teffs from all populations was compared to transcription levels of β-glucuronidase (*GUS*), a housekeeping gene, with expression reported as multiples of *GUS* expression. Two-way ANOVA found significant sources of variation associated with SHS exposure (19.26% of total variation, *P* < 0.05) and AAP exposure (56.53% of total variation, *P* < 0.05), though because a significant interaction effect was also seen (1.21% of total variation, *P* < 0.05), these results are questionable. Expression of *IFN-γ* was found to be decreased in the Teffs of FSHS subjects compared to FnSHS subjects (Figure 2B; FSHS mean ± SEM: 0.75 ± 0.05, FnSHS mean: 1.52 ± 0.11, *P* < 0.05; Hedge’s *g*: –1.67, 95% CI [−3.30, –0.07]), as well as in the Teffs of SSHS vs. SnSHS subjects (Figure 2B; SSHS mean ± SEM: 2.25 ± 0.17, SnSHS mean: 3.53 ± 0.07, *P* < 0.05; Hedge’s *g*: –2.96, 95% CI [−4.26, –1.79]). Average *IFN-γ* expression in Teffs was significantly lower in FSHS subjects than in SSHS subjects (*P* < 0.05; Hedge’s *g*: –5.17, 95% CI [−7.8, –2.75]). Expression was also significantly lower in FnSHS subjects vs. SSHS subjects (*P* < 0.05) as well as vs. SnSHS subjects (*P* < 0.05; Hedge’s *g*: –3.89, 95% CI [−5.50, –2.37]). The combined effect size of AAP + SHS (FSHS vs. SnSHS) was calculated to be −7.74 (95% CI [−10.80, –4.87]).

Linear regression analysis was performed to examine the association between methylation and transcription levels of *IFN-γ* in Teffs. Within the FSHS, FnSHS, and SSHS groups, no significant relationship between methylation and transcription levels was evident (data not shown); a significant positive association existed within the SnSHS group (Spearman *r* = 0.38, *P* < 0.05, *R*^*2*^ = 0.16, data not shown). Within the Fresno group, there was a significant negative association between methylation and expression levels of *IFN-γ* in Teffs (Spearman *r* = −0.67, *P* < 0.05; *R*^*2*^ = 0.27; data not shown); no significant correlation was seen in the Stanford group (Spearman *r* = −0.08, *P* = n.s.; *R*^*2*^ = 0.20; data not shown). A significant negative association between methylation and transcription levels was seen in both the SHS and nSHS groups (including members from both Fresno and Stanford, Figure 2C and 2D; SHS Spearman *r* = −0.36, *P* < 0.05, *R*^*2*^ = 0.20; nSHS Spearman *r* = −0.58, *P* < 0.05, *R*^*2*^ = 0.38); the same held true when all subjects were analyzed (Figure 2E; Spearman *r* = −0.75, *P* < 0.05; *R*^*2*^ = 0.56).

Linear regression analyses comparing age and methylation levels and age and transcript levels were also performed to determine if age was a confounding factor, but no significant associations between age and percent methylation or transcript levels of *IFN-γ* were detected (data not shown). Additionally, the average percent methylation and average transcript levels of *IFN-γ* for each gender were compared, and no significant difference in methylation was found between males and females within either group.

### *Foxp3* in Tregs

**Figure 3:**
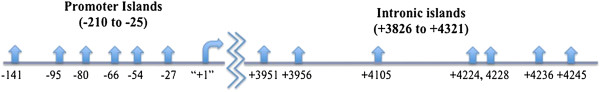
**Diagrammatic representation of Foxp3 promoter and intronic regions [NM_014009: NCBI].** Vertical arrows indicate cytosine neighboring guanine (CpG) dinucleotide sequences that can be potentially methylated. The position of each CpG dinucleotide site is illustrated in relation to the transcription start, which is indicated as +1 bp (base pair).

**Figure 4:**
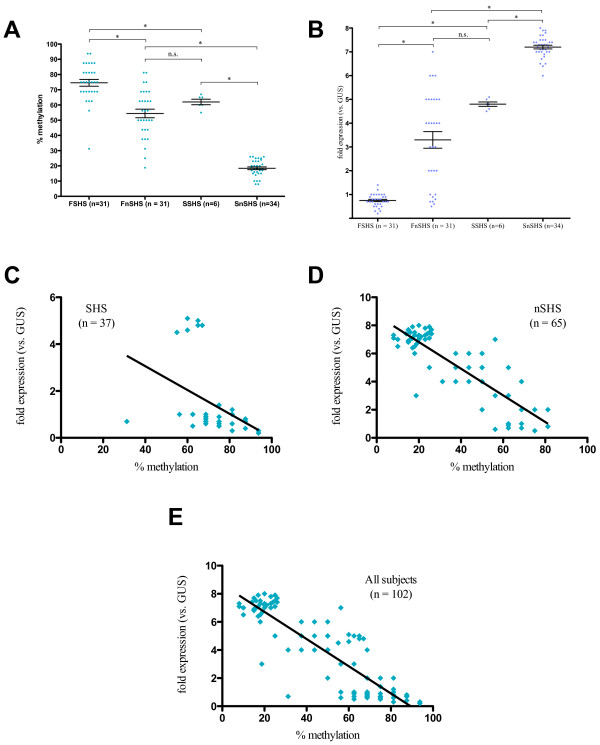
**Methylation and expression of *Foxp3 *in T regulatory cells (Tregs) of all subjects. A**. Comparison of overall methylation of examined cytosine nucleotide neighboring guanine (CpG) sites in *Foxp3* in Tregs between Fresno subjects exposed to secondhand smoke (SHS) (FSHS) (n = 31), Fresno subjects not exposed to SHS (FnSHS) (n = 31), Stanford subjects exposed to SHS (SSHS) (n = 6), and Stanford subjects who were not exposed (SnSHS) (n = 34) groups. Methylation is reported as percent methylated CpG sites out of 13 total examined CpG sites; the cutoff for positive methylation at each site was set at 70% of all sequencing reactions. Error bars represent standard error of the mean (SEM). **B**. Comparison of expression of *Foxp3* in Tregs between FSHS, FnSHS, SSHS, and SnSHS groups. Expression is reported as fold expression relative to the expression of the housekeeping gene beta-glucuronidase (*GUS*). Error bars represent SEM. **C**. Linear regression analysis of *Foxp3* expression in Tregs vs. % methylation of *Foxp3* from all SHS subjects. **D**. Linear regression analysis of *Foxp3* expression in Tregs vs. % methylation of *Foxp3* in all nSHS subjects. **E**. Linear regression analysis of *Foxp3* expression in Tregs from all subjects vs. % methylation of *Foxp3*. *P < 0.05; *n.s.*, not significant.

Average percent methylation of 13 CpG sites in the promoter and intron region of *Foxp3* (Figure 3) in Tregs was compared between all four groups. As with analysis of *IFN-γ*, sites were considered positively methylated if they were found to be methylated in 70% or more of reactions, and percent methylation was calculated for each individual by dividing the number of methylated CpG sites by the total number of CpG sites examined. Two-way ANOVA found significant effects on variation associated with SHS (39.08% of total variation, *P* < 0.05) and AAP (22.74% of total variation, *P* < 0.05), but a significant interaction effect was also seen (5.28% of total variation, *P* < 0.05). Average methylation of *Foxp3* regulatory sites in the Tregs of FSHS subjects was significantly greater than in the Tregs of FnSHS subjects (Figure 4A; FSHS mean ± SEM: 74.60% ± 2.24%, FnSHS mean: 54.44% ± 2.83%, *P* < 0.05; Hedge’s *g*: 1.40, 95% CI [1.22, 1.62]); the same held true for *Foxp3* in the Tregs of SSHS subjects vs. SnSHS subjects (Figure 4A; SSHS mean ± SEM: 62.0% ± 1.80%, SnSHS mean: 18.41% ± 0.09%, *P* < 0.05; Hedge’s *g*: 8.26, 95% CI [7.71, 9.15]). Methylation of *Foxp3* in Tregs was significantly increased in FSHS subjects compared to SSHS subjects (Figure 4A; *P* < 0.05; Hedge’s *g*: 1.05, 95% CI [0.89, 1.26]), as was methylation of *Foxp3* in the Tregs of FnSHS subjects vs. SnSHS subjects (Figure 4A; *P* < 0.05; Hedge’s *g*: 3.09, 95% CI [2.76, 3.50]). There was no significant difference between average methylation of *Foxp3* in Tregs of FnSHS and SSHS subjects (Figure 4A; *P* = 0.27). The effect size of SHS in combination with AAP (FSHS vs. SnSHS) was calculated to be 5.89 (95% CI [5.45, 6.47]).

Transcription of *Foxp3* in Tregs from all populations was compared with transcription levels of *GUS* to obtain fold expression levels. Average fold expression was compared between groups. Two-way ANOVA found significant effects associated with SHS (20.42% of total variation, *P* < 0.05) and AAP (52.77% of total variation, *P* < 0.05), with no significant interaction between exposure effects (0.02% of total variation, *P* = 0.80). Expression of *Foxp3* was found to be significantly decreased in the Tregs of FSHS subjects compared to FnSHS subjects (Figure 4B; FSHS mean ± SEM: 0.75 ± 0.05, FnSHS mean: 3.30 ± 0.35, *P* < 0.05; Hedge’s *g*: –1.81, 95% CI [−3.26, –0.40]); the same was true of *Foxp3* expression in Tregs of SSHS subjects vs. SnSHS subjects (Figure 4B; SSHS mean ± SEM: 4.8 ± 0.09, SnSHS mean: 7.2 ± 0.08, *P* < 0.05; Hedge’s *g*: –5.48, 95% CI [−6.88, –4.3]). *Foxp3* expression in Tregs was also significantly decreased in FSHS subjects vs. SSHS subjects (Figure 4B; *P* < 0.05, Hedge’s *g*: –15.00, 95% CI [−20.10, –10.60]) and in FnSHS subjects vs. SnSHS subjects (Figure 4B; *P* < 0.05, Hedge’s *g*: –2.78, 95% CI [−3.72, –1.91]). No significant difference in *Foxp3* expression was seen between Tregs of FnSHS and SSHS subjects (Figure 4B; *P* = 0.08). Effect size of SHS in combination with AAP (FSHS vs. SnSHS) was calculated to be −16.9 (95% CI [−22.00, −12.70]).

Linear regression analysis examining methylation of *Foxp3* vs. expression levels in Tregs found a significant negative association in FnSHS subjects (Spearman *r* = −0.63, *P* < 0.05; *R*^2^ = 0.34; data not shown) but none in FSHS subjects (data not shown). Within the Stanford groups, a significant positive association between methylation and expression levels was seen in the SnSHS group (Spearman *r* = 0.48, *P* < 0.05, *R*^*2*^ = 0.11; data not shown) and no association was seen in the SSHS group (data not shown). A significant negative association between methylation and expression of *Foxp3* in Tregs was seen when data from all Fresno subjects was analyzed (Spearman *r* = −0.67, *P* < 0.05; *R*^*2*^ = 0.46; data not shown); no association was seen when all Stanford subjects’ data were analyzed (data not shown). Both the SHS and nSHS groups (which included members from both the Fresno and Stanford cohorts) showed a significant negative association between *Foxp3* methylation and expression in Tregs (Figure 4C and 4D, SHS: Spearman *r* = −0.54, *P* < 0.05, *R*^*2*^ = 0.17; nSHS: Spearman *r* = −0.71, *P* < 0.05, *R*^*2*^ = 0.72). When methylation and transcription levels of *Foxp3* were examined in all subjects, there was a significant negative association (Figure 4E; Spearman *r* = −0.84, *P* < 0.05, *R*^2^ = 0.77).

As with *IFN-γ*, linear regression analyses comparing age and methylation levels and age and transcript levels were also performed, but no significant associations between age and percent methylation or expression level of *Foxp3* were seen (data not shown). Additionally, to determine if gender might be a confounding factor, the average percent methylation and average transcript levels of *Foxp3* for each gender were compared, and no significant difference in methylation was found between males and females within either group (data not shown).

A summary of these results can be found in Table 3.

**Table 3:** **Methylation and expression of *IFN-γ *in T effectors and of *Foxp3 *in T regulatory cells for all subjects**

**Parameter**	**Fresno SHS**	**Fresno nSHS**	**Stanford SHS**	**Stanford nSHS**
	**(n = 31)**	**(n = 31)**	**(n = 6)**	**(n = 34)**
***IFN-γ *methylation in Teffs (out of 6 CpG sites)**	62.10% ± 2.12%	41.29% ± 1.94%	46.67% ± 2.47%	24.85% ± 5.80%
***IFN-γ *expression in Teffs (fold expression vs. *GUS*)**	0.75 ± 0.05	1.52 ± 0.11	2.25 ± 0.17	3.53 ± 0.07
***Foxp3 *methylation in Tregs (out of 13 CpG sites)**	74.60% ± 2.24%	54.44% ± 2.83%	62.0% ± 1.80%	18.41% ± 0.09%
***Foxp3 *transcription in Tregs (fold expression vs. *GUS*)**	0.75 ± 0.05	3.29 ± 0.35	4.8 ± 0.09	7.2 ± 0.08

*IFN-γ*, interferon-gamma; *Foxp3*, forkhead box transcription factor 3; SHS, exposed to secondhand smoke; nSHS, not exposed to secondhand smoke; *GUS*, beta-glucuronidase; Teffs, T effector cells; Tregs, T regulatory cells. Means are reported ± SEM.

## Discussion

Our study examined whether exposure to SHS both alone and in combination with AAP was associated with increased methylation and decreased expression of *IFN-γ* in Teffs and of *Foxp3* in Tregs. Our assessment of SHS exposure, based on parent questionnaires, was dichotomous: subjects were determined to have either been exposed to SHS or not. The validity of questionnaires asking about parental smoking and smoking in the home to determine pediatric exposure to SHS has been demonstrated previously, with questionnaire-determined SHS exposure confirmed by cotinine analysis [1,34,35]. To confirm SHS exposure in some subjects, we performed urinary cotinine analysis on a subset of subjects from our Fresno cohort, and found that there was a significantly higher level of urinary cotinine in our questionnaire-determined SHS vs. nSHS subjects.

In both cohorts, we found that subjects exposed to SHS showed a significantly greater percentage of methylation of CpG sites in the *IFN-γ* promoter in their T effector cells when compared to the Teffs of subjects not exposed to SHS (Figure 2A). Consequently, expression of *IFN-γ* was significantly decreased in the Teffs of SHS subjects in comparison with the Teffs of nSHS subjects in both cohorts (Figure 2B). Interestingly, while there was a significant increase in methylation and decrease in expression of *IFN-γ* in Teffs associated with AAP exposure (that is, between the FSHS and SSHS groups and between the FnSHS and SnSHS groups), we saw no significant difference in the levels of methylation associated with AAP exposure alone (FnSHS) vs. SHS exposure alone (SSHS), though we did see a significant difference between expression levels of *IFN-γ* in both groups, with decreased expression in Teffs from FnSHS subjects vs. Teffs from SSHS subjects. When we performed linear regression analysis to determine the association between frequency of methylation and expression levels of *IFN-γ*, our results were inconsistent within the individual groups (FSHS, FnSHS, SSHS, SnSHS; data not shown), but we found a significant negative association between methylation and expression of *IFN-γ* in Teffs in both the SHS and the nSHS groups (Figures 2C,D) that was also seen when data from all subject sizes were analyzed (Figure 2E), with increases in *R*^*2*^ values also seen as population size increased. The disparity at the smaller group level therefore seems to be due to statistics and sample size rather than to any true difference between populations, as increasing the sample size led to more consistent and significant data. We can therefore conclude that there is in fact a negative association between CpG methylation of the *IFN-γ* promoter regions and expression of *IFN-γ* in Teffs, a result that matches those of previous studies [20-22]. Furthermore, as IFN-γ is the major Th1 cytokine [24], our finding of increased methylation and decreased expression of *IFN-γ* in Teffs of subjects exposed to SHS and subjects exposed to AAP, as well as subjects exposed to both, suggests that SHS and AAP impair normal Th1 function both independently and in conjunction with each other. However, as we did not assess Teff or Th1 function, we cannot verify this.

Similarly, we found that subjects exposed to SHS had a significantly greater percentage of methylation of CpG sites in the promoter and intron regions of *Foxp3* in their regulatory T cells when compared to *Foxp3* in the Tregs of nSHS subjects, and that expression of *Foxp3* in the Tregs of SHS subjects was therefore significantly decreased. The same was true of subjects exposed to AAP vs. subjects not exposed to AAP (Figure 4A,B). Linear regression analysis showed a significant negative association between methylation of the regulatory regions of *Foxp3* and expression of the gene within both the SHS population (Figure 4C) and the nSHS population (Figure 4D), as well as within the entire population (Figure 4E), despite inconsistent associations in the individual groups; as with *IFN-γ*, these inconsistencies were likely due to decreased sample size having decreased statistical power. Stable expression of *Foxp3* is necessary for normal Treg function [25]; our results therefore suggest that exposure to SHS or AAP alone, and to both SHS and AAP, may impair Treg function via methylation and consequent decreased expression of *Foxp3*. While we did not assess Treg function, a previous study has shown that increased methylation of *Foxp3* leads to decreased Treg activity, supporting this conclusion [4]. The significantly increased IgE concentrations in SHS subjects vs. nSHS subjects also provide positive evidence for decreased Treg function, as decreased Treg function leads to reduced immunosuppression of Th2 cells, leading to increased levels of the Th2 cytokines IL-4 and IL-13, which induce increased B cell production of IgE [36]. However, as this association was only seen in FSHS subjects vs. nSHS subjects, it would appear that changes in IgE are more influenced by the combination of AAP and SHS exposure rather than AAP or SHS alone.

As expected, we found that both AAP alone and SHS alone were associated with significant increases in methylation and decreases in expression of both *IFN-γ* in Teffs and *Foxp3* in Tregs (Figures 2A,B, 4A,B). Of greater interest is the interaction between these exposures and their combined and relative effects upon the methylation and expression of these two genes. Our finding that there is no significant difference between the levels of methylation of *IFN-γ* in Teffs and the levels of methylation and expression of *Foxp3* in Tregs in subjects exposed to SHS alone vs. subjects exposed to AAP alone suggests that neither is more significantly associated with methylation than the other. However, when looking at calculated effect sizes, SHS exposure alone seems to have a greater association with methylation and transcription of both genes than AAP exposure alone; in every measurement except that of *IFN-γ* expression levels, the SHS effect size is at least twice as large as that of the AAP effect size. (It should, however, be noted that because our SHS-only sample size is very small, any conclusions we draw are more indicative of trends.) Additionally, when looking at relative contributions of SHS and AAP to variance of the means via two-way ANOVA, while significant effects on the total variance were associated with both exposures, the degree of the effect differed, with SHS contributing more to variation of methylation of both genes and AAP contributing more to variation of expression of both genes. The implications of these findings are as yet unclear and require more data regarding timing and relative degree of AAP and SHS exposures in relation to each other.

Beyond their individual effects, AAP and SHS also appear to have a greater association with altered methylation and expression of *Foxp3* and *IFN-γ* in Tregs and Teffs when combined, as can be seen by the increased methylation and decreased expression of these genes in the FSHS subjects (AAP + SHS) vs. the FnSHS subjects (AAP alone) and the SSHS subjects (SHS alone), as well as the increased effect size when compared with the SnSHS group. Given that AAP and SHS both share certain components (including PAH, which we know to be associated with increased methylation and decreased expression of *Foxp3* in Tregs [4,37]), this finding seems logical. The details of this interaction, however, are unclear. Looking at effect sizes for AAP and SHS on methylation and expression of *IFN-γ* suggests these the two exposures are associated with these changes in an additive fashion, as the sum of the individual effect sizes of AAP and SHS exposures alone is close to the effect size of AAP + SHS for both *IFN-γ* methylation and transcription. However, two-way ANOVA found significant interaction between AAP and SHS on variation of mean transcription levels of *IFN-γ*, which would suggest a synergic association. *Foxp3* effect sizes also suggest a synergic association rather than a perfectly additive one, but do not present a clear picture: the effect size of AAP + SHS is nearly twice as large as the sum of the individual effect sizes of AAP and SHS on *Foxp3* transcription, while the effect size of AAP + SHS on *Foxp3* methylation is nearly half the sum of the individual effect sizes of the exposures. Additionally, while two-way ANOVA showed a significant interaction between AAP and SHS on the variance of *Foxp3* methylation levels, suggesting a synergic association, the interaction between the two exposures was not found to be significant with relation to *Foxp3* transcription, which would suggest an additive association.

In part, our findings conflict with previous findings that acute SHS exposure contributes more significantly to clinical symptoms of asthma than does AAP exposure [10], though it should be noted that in the Rabinovitch *et al*. study, there was some overlap between measures, as the study looked at PM_2.5_ as the measure of AAP, which can also be found in SHS. However, our study only assessed if subjects had been exposed to SHS or not, without looking at the time of most recent exposure, so a direct comparison between our study and the Rabinovitch *et al*. study is not appropriate (indeed, our cotinine assay results would suggest that a number of our SHS subjects had not experienced acute SHS exposure in relation to sample collection time). Additionally, we have not yet looked at any functional assays or clinical assessments to determine if the differences in methylation and expression of our two genes of interest are borne out in the immunophenotypes of the subjects. At the same time, our findings are intriguing, showing that the combination of AAP and SHS is more significantly associated with methylation and expression of *IFN-γ* in Teffs and *Foxp3* in Tregs than independent exposure to these compounds, and thus impairs normal immunosuppressive functions to a greater degree than would be seen with either AAP or SHS alone.

Our findings, to our knowledge, are novel in that they show a significant relationship between SHS and AAP and hypermethylation and therefore decreased expression of *IFN-γ* and *Foxp3*, two genes crucial to the suppression of the asthmatic and allergic responses. SHS exposure is associated with increased comorbidity of asthma and allergic rhinitis, but the mechanisms by which these occur are still unclear. Several studies have implicated SHS and AAP in epigenetic changes to the human genome, but the list of specific genes impacted is incomplete [11,13,16,17]. We chose to study *IFN-γ* and *Foxp3* both because of their significance to immunosuppression and because previous studies have associated exposure to ambient air pollution and/or allergens with hypermethylation of these genes [4,23,28,38]. The results of our study therefore imply that at least one way in which SHS and AAP may contribute to asthma and allergies is via epigenetic modifications that disrupt the normal function of genes crucial to Teffs and Tregs.

Our findings are supported by evidence that exposure to cigarette smoke or AAP is associated with decreased numbers and function of Teffs and Tregs [4,39-43]. Decreased presence and activity of Teffs and Tregs have also been implicated in the prevalence and severity of asthma, with various studies finding skewing of Th2/Th1 ratios in favor of Th2 activity and of Th17/Treg ratios in favor of Th17 activity in patients with moderate to severe asthma [44-47]. We can thus infer that increased exposure to SHS and AAP leads to hypermethylation of *IFN-γ* in Teffs and *Foxp3* in Tregs, leading to decreased expression of these genes, causing impaired function of Teffs and Tregs that therefore leads to increased severity and prevalence of asthma and allergies. This is supported by our finding of elevated levels of IgE in FSHS-exposed subjects vs. FnSHS subjects. However, since this difference between IgE levels in SHS and nSHS subjects was not seen in the Stanford cohort, IgE and the allergic response appears to be more associated with AAP exposure than with SHS exposure (two-way ANOVA, however, showed a significant SHS effect of 6.57% (*P* < 0.05) vs. a significant AAP effect of 3.53% (*P* < 0.05)).

Our data do not provide significant evidence that SHS or AAP and impaired Teff and Treg function are necessarily causative in the development of asthma or allergic rhinitis. Complete clinical data is needed to examine the link between SHS and AAP and these diseases.

In addition to examining the independent associations of AAP and SHS with the methylation and expression of our candidate genes, we looked to see if we could begin to determine the relative impacts of the two exposures on mechanisms of the immune system. A previous study conducted on patients from Denver looked at the relative impact of the two exposures on clinical symptoms, and demonstrated that recent SHS exposure decreased the impact of AAP on asthmatic exacerbations while simultaneously increasing asthmatic exacerbations [10]. Similarly, we found that subjects exposed to SHS in addition to AAP had increased methylation and decreased expression of *Foxp3* in their Tregs and of *IFN-γ* in their Teffs when compared with the Tregs and Teffs of subjects exposed to AAP alone, suggesting that SHS is associated with aggravation of the effects of exposure to AAP.

At the same time, we did not find a significant difference in *IFN-γ* methylation in Teffs or in *Foxp3* methylation and expression in Tregs between subjects exposed to AAP alone and subjects exposed to SHS alone, suggesting that the SHS + AAP association is a combinatory one. Further study is needed to examine functional consequences of the epigenetic modifications of *IFN-γ* and *Foxp3* on Teffs and Tregs, respectively, as well as to see if these relative associations hold true for other genes. SHS and AAP are not the only factors known to influence asthma, nor are *IFN-γ* and *Foxp3* the only genes relevant to the development and exacerbation of the disease [2,13,48-50]; other factors, both environmental and genetic, are currently under investigation. We have, to date, examined other indoor environmental exposures such as pollen, dust mites, endotoxin, and mold, but found the greatest differences associated with SHS exposure or lack thereof. Going forward, we will continue to assess these other exposures.

Moreover, SHS and AAP are not the only factors potentially impacting DNA methylation. Previous studies have demonstrated that certain genes are either hyper- or hypomethylated with age [51-54], but no study to date of which we know has assessed DNA modification of *Foxp3* or *IFN-γ* with respect to age in different T cell subsets. When we performed linear regression of age vs. methylation for both genes, we found no association. Gender has also been implicated in differences in methylation [15,54,55], but we found no significant difference in methylation of either gene in any group associated with gender, and when controlling for gender, obtained no difference in our findings. These results suggest that any contributions of age and gender to the methylation of *Foxp3* and *IFN-γ* in Tregs and Teffs, respectively, are relatively small in relation to the impacts of SHS and/or AAP. Future studies should, however, continue to monitor age and gender.

One further confounding factor is suggested by the significantly increased total IgE seen in FSHS patients vs. nSHS patients. Increased IgE levels are frequently associated with increased allergic severity [56], which is in turn often associated with increased methylation and decreased expression of *Foxp3* in Tregs and of *IFN-γ* in Teffs [21,25]. Additionally, studies have found an association between SHS exposure and increased IgE levels [57]. This suggests that SHS might affect methylation by increasing allergic severity. Overall allergic severity for a given subject, however, is difficult to determine [58,59]; we did perform skin tests and administer questionnaires concerning allergic rhinitis, and found that subjects with a total IgE >25 kU/L reported symptoms of allergic rhinitis. Given our small sample size, however, we can only infer at this point that total IgE levels may be indicative of clinical atopy. Additionally, given the measures we examined, we did not see any significant difference in allergic severity between SHS and nSHS subjects; the most significant difference between the populations was their SHS exposure status, suggesting that SHS influences methylation directly rather than via an intermediate. It should also be noted that, while increased total IgE is frequently correlated with increased allergic severity, this does not always hold true [60].

Further limitations of our study include our small sample size, as well as the lack of temporality, given that this is a cross-sectional analysis.

Future studies should aim to correlate our findings of genetic differences with phenotypic differences. Teff and Treg functional assays should be performed to determine if the observed changes in methylation and expression of *IFN-γ* and *Foxp3*, respectively, impair the normal behavior of these cells. Immunophenotype analysis to evaluate the relative numbers and activity levels of Teffs and Tregs between SHS and nSHS subjects (with and without AAP) from this study should also be performed. Moreover, data from more subjects should be added to our analysis to increase the potential significance of our findings, and data should be collected at multiple time points to examine the temporality of these methylation associations, if any exist. Along these lines, it will also be important to gain a more nuanced picture of the association between these exposures and methylation of these genes, looking at such factors as timing of exposure, duration and extent of exposure, prenatal vs. postnatal exposure (particularly with respect to SHS), and whether this methylation association persists after the exposure has been removed from the environment. Additionally, clinical allergy and asthma profiles and their potential relation to SHS exposure, both with and without AAP, should be examined. To better understand the mechanisms underlying expression of both *IFN-γ* and *Foxp3*, methylation at individual CpG sites should also be analyzed. Finally, it is necessary to study other genes important in allergies and asthma that could be epigenetically modified, with repeated measurements performed for these genes as well as *IFN-γ* and *Foxp3* to assess the durability of these changes over time.

## Conclusions

We show that there are significant epigenetic differences in two key genes known to be associated with immunological changes in allergy and asthma between groups of children exposed to SHS (both in combination with AAP and not) and a group of controls. Our data demonstrate a significant association between SHS and AAP and hypermethylation and decreased transcription of *IFN-γ* and *Foxp3* in T effector and T regulatory cells, respectively, in comparison with SHS exposure alone and AAP exposure alone. This suggests a possible mechanism by which SHS and AAP may be associated with the development and progression of asthma and allergies, as decreased levels of expression of these genes have been linked with increased prevalence and severity of asthma and allergic disease [39-47]. Further studies are needed to elucidate the precise relationship between SHS and AAP and these epigenetic changes, as well as to correlate the genetic profiles of these subjects with their clinical and cellular profiles.

## Methods

### Participants

Participants were recruited from the Fresno Unified School District and screened at the Fresno Field Office (work supported by the NIEHS/EPA P20 Children’s Environmental Health Center). A total of 62 participants aged between 11 and 18 years, all residents of Fresno for at least 9 years, provided informed consent and were enrolled into this study. All of these individuals were selected for detailed study regarding the changes in methylation of *IFN-γ* and *Foxp3* associated with SHS. In addition, we recruited a control group (Stanford cohort) which consisted of 40 subjects from Palo Alto, California, a region with relatively low ambient air pollution (California Air Resources Board (CARB), http://www.arb.ca.gov). All control subjects had lived in Palo Alto for at least 6 years and lived at least 800 m from any major highways. All study components were approved by the Institutional Review Board (IRB) of Stanford University. Urine and blood samples were collected on all participants. Blood and urine were stored according to published procedures. In short, urine was initially stored at −20°C before being transferred to a −80°C freezer. Peripheral blood mononuclear cells (PBMCs) were extracted from blood via the ficoll procedure, slowly frozen to −80°C overnight, and then stored in liquid nitrogen. Plasma was extracted from the blood at the same time and stored at −80°C. Plasma IgE levels for subjects at the time of blood draw were determined. PBMCs were further purified for CD4^+^CD25^neg^ Teff cells and, separately, for CD4^+^CD25^hi^ Treg cells (FACS Aria, BD Biosciences). *IFN-γ* and *Foxp3* methylation data were obtained for all enrolled subjects.

### Measures of exposure to AAP

AAP was measured and individual estimate exposures were determined for all Fresno subjects. All subjects were monitored for PM_2.5_, PM_10_, PAH, and ozone through methods reported previously [4], using data from CARB. Briefly, daily pollutant exposures were assigned to subjects on the basis of measurements from the nearest CARB monitoring site (Fresno subjects: First Street, Fresno, CA; Stanford subjects: Redwood City, CA), using the following measures: 8-h daily maximum ozone (O_3_) concentration; and 24-h average concentrations of PM_2.5_, PM_10_, and PAH. For Fresno subjects, daily First Street data were used directly for all pollutants except PAHs. Each subject’s individual PAH exposure estimate for a cumulative 12 months of exposure (the 12 months prior to the clinic visit in which the questionnaire for SHS was administered) was calculated based on land use regression analyses, which formalize the relationship between measurement site and local environmental variables such as traffic flow and land cover and allow for consideration of temporal and spatial variation [61,62], using measurements taken at subjects’ homes. AAP exposure analysis was performed by collaborators from UC Berkeley and the Fresno field office. Because this was cumulative over 12 months, seasonal effects were not taken into account. These data and a more detailed description of these methods can be found in our previously published paper [4].

### Measures of exposure to SHS

All subjects were monitored for secondhand smoke exposure via questionnaires asking the parents: ‘Do you currently smoke?’ and ‘Does anyone who currently spends time with the child smoke?’ On the basis of the responses to these questionnaires, 62 subjects from the Fresno cohort, 31 exposed to SHS and 31 not, were chosen for *IFN-γ* and *Foxp3* methylation study. To confirm the accuracy of our questionnaire in determining SHS exposure, an enzyme-linked immunosorbent assay (ELISA) for measures of cotinine in the urine was performed as a proof of concept in a small subset of FSHS (n = 16) and FnSHS (n = 6) patients using a modified High Sensitivity Cotinine EIA assay (Salimetrics, State College, PA, USA) with a limit of detection of 0.1 ng/mL. Based on Thompson *et al*. [63], a cutoff of 7 ng/mL was considered positive for very recent exposure to SHS.

### Purification of T cells

CD4^+^ T cells were enriched by incubation with the negative selection CD4^+^ isolation kit from Stem Cell Technologies (Vancouver, BC, Canada). We centrifuged 1:1 heparinized whole blood phosphate-buffered saline (PBS) with 1% BSA at 450 *g* for 40 minutes over Ficoll-Hypaque density gradients. The CD4^+^ T-cell-containing PBMC layer was collected and washed twice with 10 mL PBS at 4°C. After centrifugation at 250 *g* for 15 minutes, cells were resuspended in PBS in flow cytometry staining tubes. Live/Dead staining (Molecular Probes/Invitrogen, Grand Island, NY, USA) was used in all samples. Live cells were identified by the intracellular conversion of a calcein ester to free calcein (intensely fluorescent in the green spectrum), and dead cells were identified by the red staining of internal nucleic acids by ethidium homodimer. Subsequently, the live cell fraction was stained with CD4-fluorescein isothiocyanate (clone SK3; BD Biosciences, San Jose, CA, USA), CD25-phycoerythrin (clone 4E3; Miltenyi Biotec, Auburn, CA, USA) and CD127 (IL-7 receptor α-chain) allophycocyanin (clone SB199; BioLegend, San Diego, CA, USA) antibodies and sorted by flow cytometry for live CD4^+^CD25^hi^CD127^lo/-^ Treg cells (FACS Aria; BD Biosciences, San Jose, CA). CD127 depletion occurred to ensure separation of activated conventional T cells from stable Treg-cell populations, which allowed for the isolation of highly enriched Foxp3^+^ cells. Live non-Treg (CD4^+^CD25^-^) cells (subsequently called conventional effector CD4^+^ T cells or Teffs) were flow-sorted simultaneously. Past experiments with multicolor flow-cytometry staining for CD4^+^CD25^hi^CD127^lo^ (Treg) cells and CD4^+^CD25^-^ (Teff) cells have demonstrated that each cell population was routinely > 95% pure. The Treg and Teff cells were incubated in RPMI-1640 media, 10% fetal bovine serum (FBS), and 1% L-glutamine after purification for 2 h before undergoing further experiments. All experiments were controlled for live cell number.

### DNA isolation and sodium bisulphite conversion

Genomic DNA was isolated from purified Teff and, separately, Treg cells, from each subject’s samples using a DNeasy Mini Kit (Qiagen, Valencia, CA, USA) according to the manufacturer’s instructions and controlling for cell number. Genomic DNA was bisulphite-treated using published procedures [4]. Briefly, 1 to 2 *μ*g of genomic DNA in 45*μ*L of nuclease-free water was denatured at 42°C for 20 minutes with 5*μ*L of freshly prepared 3 M sodium hydroxide. Denatured DNA was incubated with freshly prepared sodium bisulphite (saturated) and hydroquinone solution in the water bath at 55°C for 16 h. The bisulphite-converted DNA was purified using a Wizard DNA Clean-up Column (Promega, Madison, WI, USA) and then desulphonated by incubation with 5.5*μ*L of a 3 M NaOH solution at 37°C for 20 minutes. The bisulphate-treated DNA was finally precipitated with ethanol, and then redissolved in 25 *μ*L of 1 mM Tris-Cl pH 8.

### *IFN-γ* bisulphite-specific polymerase chain reaction (PCR) and pyrosequencing

The detailed information of primers used in the bisulphite-specific PCR can be found in Table 4. For methylation analysis, five amplicons, amplified using a HotStar Taq kit (Qiagen, Valencia, CA, USA), included a total of six CpG sites within the proximal promoter region of *IFN-γ* (Figure 1). Direct quantification of methylated vs. unmethylated cytosine nucleotides for each analyzed CpG site present in the amplicons was determined by pyrosequencing with the PSQ HS 96 Pyrosequencing System (Qiagen) and Pyro Gold CDT Reagents (Qiagen) as described previously [64]. Positive methylation thresholds for each site were set at 70% cytosine methylation or more. In each pyrosequencing assay, one amplicon was used for sequencing; the corresponding sequencer can be found in Table 4. Internal controls for bisulphite conversion efficiency were included in each pyrosequencing assay. A genomic sequence that is artificially methylated on all its CpG dinucleotides (catalogue number S7821, Millipore, Billerica, MA, USA) was also used in the bisulphite conversion, PCR, and pyrosequencing with the primers and sequencers mentioned above as a technical control.

**Table 4:** **Oligonucleotides used for bisulphite-specific polymerase chain reaction and pyrosequencing of *IFN-γ***

CpG Site*	**Details**	**Sequences**
−295	Forward	5′-[Biotin]TTTGTAAAGGTTTGAGAGGTTTTAGAAT-3′
	Reverse	5′-CAAACCCATTATACCCACCTATACCA-3′
	Sequencer	5′-TTTTATACCTCCCCACTT-3′
−186	Forward	5′-TTAGAATGGTATAGGTGGGTATAATGG-3′
	Reverse	5′-[Biotin] TATTATAATTAAAATTTCCTTTAAACTCCT-3′
	Sequencer	5′-GGGTATAATGGGTTTGTT-3′
−54	Forward	5′-GGGTTTGTTTTATAGTTAAAGGATTTAAGG-3′
	Reverse	5′-[Biotin] AATCAAAACAATATACTACACCTCCTCTAA-3′
	Sequencer	5′-TATTTTATTTTAAAAAATTTGTG-3′
+122~	Forward	5′-[Biotin] TTTTGGATTTGATTAGTTTGATATAAGAA-3′
+128	Reverse	5′-AAAACCCAAAACCATACAAAACTAAAA-3′
	Sequencer	5′-CTAAAAAACCAAAATATAACTTAT-3′
+171	Forward	5′-[Biotin] TTTTGGATTTGATTAGTTTGATATAAGAA-3′
	Reverse	5′-CATTTTCAACCACAAACAAATACTATTAA-3′
	Sequencer	5′-ACAACCAAAAAAACCC-3′

*CpG sites indicate nucleotide position in relation to transcription start. Symbols – and + indicate upstream and downstream of transcription start, respectively. CpG, cytosine nucleotide neighboring guanine.

### *Foxp3* bisulphite-specific PCR and pyrosequencing

Identification of *Foxp3* CpG loci of interest (Figure 3) was based on our previous studies [4] of two regions where we found increased methylation of the 8 CpG islands in the promoter region and of the 13 CpG islands in the intronic regions. Demethylation of these two regions was associated with stable *Foxp3* expression in memory Tregs. Partial methylation was associated with unstable *Foxp3* expression in Tregs. In contrast, the promoter and intronic regions of *Foxp3* in conventional CD4^+^ T cells are methylated. Table 5 gives detailed information about the primers used in the bisulphite-specific PCR.

**Table 5:** ***Foxp3 *pyrosequencing/primer design**

	***Foxp3 *promoter**	***Foxp3 *intronic region**
**Forward**	5′-CAAATTCAGAGTATTAGTTCTTTTCCTCTTT−3′	5′-TTGGGTTAAGTTTGTTGTAGGAT−3′
	
**Reverse**	5′-[Biotin]-GTGGCATGTCGCACCAAAAAGAAGAG−3′	5′-[Biotin]- ACCCCCCACTTACCCAAATTTT−3′
	
**Sequencing**	5′-AAAGGCAAATTCA-3′	5′-GTAGGATAGGGTAGTTAG-3′
**Description**	6 CpG sites*	7 CpG sites*
	(-141, -95, -80, -66, -54, -27)	(+3951, +3956, +4105, +4224, +4228, +4236, +4245)

*CpG sites indicate nucleotide position in relation to transcription start. Symbols – and + indicate upstream and downstream of transcription start, respectively. CpG, cytosine nucleotide neighboring guanine.

To display the methylation data for both genes, we first obtained pyrosequencing data as to whether a particular CpG site was methylated (a threshold of 70% or higher methylation frequency was considered a methylated CpG site) in purified T cells that were controlled for cell number. Then, to calculate a separate percentage of CpG sites for a given locus, we divided the total number of methylated CpG sites (numerator) for a specific genetic locus (either *Foxp3* or *IFN-γ*) by the total CpG sites sequenced (denominator) and used this percentage in our figures presented here.

CpG islands were determined using NCBI gene searches, BLAST for sequence information; primers were determined using PyroMark Assay Design Software (Qiagen).

### Quantitative real-time PCR

Total RNA was isolated from purified cells with the use of an RNeasy Mini Kit (Qiagen). cDNA from 500 ng of total RNA was synthesized using an Omni script Kit (Qiagen) and random declaimer primers (catalogue number 5722E, Austin, TX, USA). Real-time PCR was performed with 1*μ*L of synthesized cDNA, 12.5*μ*L TaqMan Universal PCR mix, and 1.25*μ*L 20X assay on demand gene expression assay mix (Mm00445273_m1, Applied Biosystems, Foster City, CA, USA), in a 7000 Sequence Detection System (ABI Prism, Applied Biosystems, Carlsbad, CA, USA). Each sample was performed in duplicate. The ribosomal 18S Control Reagents (part number 4308329), also from Applied Biosystems, was used as an endogenous control for data normalization. The relative quantity of *IFN-γ* and *Foxp3* mRNA was calculated against *GUS* values according to the methods published by our group [4].

### Statistical analysis

Two-way ANOVA was performed to compare the relative associations of AAP and SHS with methylation and expression, and followed by *t*-tests to compare the means of all parameters tested. Effect sizes were calculated using Hedge’s *g* and reported with the 95% confidence interval. Linear regression analysis was applied to determine the association between percent CpG methylation (averaged across all CpG sites examined for each gene) and expression levels for *IFN-γ* and *Foxp3* in Teffs and Tregs, respectively. Linear regression analyses were also performed to determine if any confounding association existed between the demographic factors of age and gender. The threshold for all statistical significance was set at *P* < 0.05. All statistical analysis was performed with the Graph Pad Prism Software (Prism Software, La Jolla, CA, USA).

## Competing interests

The authors declare that they have no competing interests.

## Authors’ contributions

AK processed samples, performed the statistical analysis, and drafted the manuscript. MAG processed samples. RLM carried out urinary cotinine analysis and edited the manuscript. CM carried out urinary cotinine analysis. OH participated in the design of the study and its statistical analysis. SKH participated in the design and coordination of the study. KCN conceived of the study, participated in its design and coordination, performed statistical analysis, and helped draft the manuscript. All authors read and approved the final manuscript.
